# Improving outcomes by empowering nurses to lead change in an acute stroke unit

**DOI:** 10.1097/NSG.0000000000000416

**Published:** 2026-06-22

**Authors:** Emma L. Hutton, Donna F. Manlangit, Billie Curren, Mymoona Vettil, Nancy Al Ali, Ehab Zayed

**Affiliations:** In the Acute Stroke Unit, Sheikh Shakhbout Medical City, Abu Dhabi, United Arab Emirates, **Emma L. Hutton** is Nurse Manager, and **Donna F. Manlangit**, **Billie Curren**, **Mymoona Vettil**, **Nancy Al Ali**, and **Ehab Zayed** are Charge Nurses.

**Keywords:** nurse leadership, patient safety, quality improvement, shared governance

## Abstract

**Background:**

There is increasing awareness of the link between nursing staff satisfaction, patient satisfaction, and clinical outcomes. Therefore, it would benefit health care organizations to critically appraise and implement cost-effective strategies and practices that address nursing staff engagement and encourage behavior that improves patient satisfaction and outcomes. The aim of this quality improvement (QI) project was to empower and inspire nurses to provide optimal, evidence-based care that enhanced the patients' experience and optimized clinical outcomes in a specialized stroke unit.

**Methods:**

The authors employed the Plan-Do-Study-Act model embedded within the Daily Team System QI framework to structure the implementation strategies for maximum effectiveness. Data measurements included NDNQI RN Satisfaction Surveys; Press Ganey Patient Satisfaction Surveys; and Nurse Sensitive Quality Indicators for falls, hospital acquired pressure injuries (HAPIs), catheter-associated urinary tract infections, and hand hygiene adherence. Analysis was descriptive and allowed for inferences to be drawn about how the implemented strategies positively impacted outcomes.

**Results:**

There were significant improvements across measured clinical outcomes for falls, HAPIs, and hand hygiene adherence. Patient satisfaction scores increased, and RN satisfaction survey results demonstrated that implemented strategies had a positive effect on nursing staff's experience and behavior.

**Conclusion:**

The changes and improvements were relatively easy to implement and were highly impactful across measured outcomes. Empowering nurses to lead quality initiatives and using a structured change management framework benefits patients and fosters a more fulfilled, effective nursing team.

## Introduction

Health care organizations are increasingly aware of the link between nursing staff satisfaction, patient satisfaction, and clinical outcomes. A significant and avoidable proportion of global health care costs are the result of poor clinical outcomes and adverse events from medical treatment.^1^ In a global economic report on health care, the World Health Organization (WHO) calls for governments to focus greater efforts on addressing patient safety, improving health care quality, increasing efficiency, and reducing costs.^2^ Therefore, it would benefit all health care professionals and leaders to critically appraise and implement cost-effective strategies and practices that mitigate adverse medical events and improve patient satisfaction and outcomes.

A common finding in most root-cause analyses of health care–related adverse events is problematic communication and inadequate leadership.^3^ The evidence overwhelmingly indicates that disengaged and dissatisfied nurses negatively impact patient satisfaction and safety.^4^,^5^ Therefore, it is imperative that nurses take the lead and drive improvement initiatives to address barriers to satisfaction and engagement. In the acute stroke unit at a tertiary referral hospital in the United Arab Emirates (UAE), nurses were inspired to improve clinical outcomes and patient satisfaction by enhancing their work environment through several unit-based initiatives. This was facilitated by structural empowerment that is the foundation of the shared governance quality improvement (QI) model.^6^,^7^

## Background

Quality and safety are the motivating forces behind a shared desire to improve health care, which requires staff who are motivated and engaged.^8^,^9^ This QI project was conducted on a 25-bed acute stroke unit in a large, 590-bed tertiary hospital in the UAE. The hospital's unit-based QI activities are data driven and evidence based. They are guided by the primary value of putting patients first through advanced, personalized care. Achieving this goal requires educating, empowering, and facilitating nurses to deliver compassionate person-centered care.^8^ The aim of the current project was to enhance the experience of nurses and patients while simultaneously improving clinical outcomes. Further, changes also needed to be cost effective and sustainable over the long term. Facilitating changes involved the application of transformational leadership strategies, a shared governance framework and the Plan-Do-Study-Act (PDSA) QI model.^10^,^11^

Transformational leadership in nursing is a leadership approach that focuses on inspiring and motivating nursing teams to exceed expectations and drive meaningful change.^8^ This leadership style emphasizes the creation of a shared vision, supports staff's personal and professional development, and actively encourages innovation and creative problem-solving. Transformational nurse leaders empower their teams through structural support strategies, such as shared governance frameworks, which enable nurses to take ownership of QI initiatives and contribute to unit-based decision-making processes.^6^,^8^,^10^,^12^ By cultivating an environment where nurses feel valued, engaged, and supported, transformational leadership directly addresses the critical link between staff satisfaction, patient safety, and clinical outcomes. This approach is effective because it fosters a culture of continuous improvement, professional growth, and person-centered care delivery that benefits both nursing staff and patients.^10^ This leadership style underpins the shared governance framework that enabled staff to bring their ideas and suggestions to the unit-based committee for consideration.^6^,^12^ Once the strategies were considered and accepted, PDSA was utilized to implement the changes.^13^

## Methods

The PDSA iterative improvement method utilizes small-scale, rapid cycles to implement and test interventions before broader implementation.^13^ PDSA is embedded within the Daily Teams System (DTS) shared governance QI framework adopted by the organization.^13^,^14^ Project implementation was conducted in accordance with each sequential stage of the PDSA cycle:

- **Plan:** Ideas generated from the DTS and baseline data were analyzed to develop objectives and actions such as improving duty schedule practices, creating awareness huddles, and so forth.^13^
- **Do:** The actions were implemented and observed for effectiveness.^13^
- **Study:** First quarter data were gathered and fully analyzed to compare results with predictions and summarize learnings.^13^
- **Act:** Adjusted the plan next cycle, increasing staff numbers to meet increased bed numbers and including the whole multidisciplinary team in the hand hygiene awareness huddles.^13^

This project measured quantitative data across three domains: clinical outcomes, patient satisfaction, and nurse satisfaction. As a QI project, it was exempt from institutional review board approval. It is reported here using the revised Standards for Quality Improvement Reporting Excellence (SQUIRE) 2.0 guidelines.^15^,^16^ Data were obtained from the National Database of Nursing Quality Indicators (NDNQI) RN Satisfaction Survey,^17^ Press Ganey Patient Satisfaction Surveys,^18^ and the hospital's Nurse Sensitive Quality Indicator (NSQI) data.

The specific clinical outcomes chosen were determined by data collected in 2024 and early 2025 that indicated these outcomes were either not meeting or barely meeting organizational benchmarks. The December 2024 results of the NDNQI RN Satisfaction Survey revealed that the overall satisfaction of staff in the unit was below the means of other non-Magnet-accredited hospitals in the region. The Press Ganey Patient Satisfaction Survey results for 2024 barely met the hospital's benchmark, and the unit recently experienced an upturn in the occurrence of falls and hospital-acquired pressure injuries (HAPIs) and a decrease in adherence with hand hygiene technique. These data were the drivers for evidence-based strategies that would improve patient safety.^4^,^12^,^19^

The nurse unit manager created five teams based on the clinical outcomes measured. Each team was led by a charge nurse and included four staff nurses. The teams included (1) catheter-associated urinary tract infection (CAUTI) prevention, (2) infection prevention and control (hand hygiene), (3) HAPI prevention, (4) fall prevention, and (5) the unit-based shared governance committee (UBC). The teams were responsible for implementation of interventions, disseminating information, conducting audits, data review, and data analysis. Staff were encouraged to submit suggestions for improvement through the DTS. The UBC then reviewed the suggestions and decided on those to be included in action plans. Furthermore, the manager implemented strategies to improve nurse satisfaction and clinical outcomes (see *Implemented actions including the domain and initiator*).

### Measurements

The hospital collects data and metrics on clinical outcomes which are available to all staff on the hospital's infection control and quality intranet portals. Data for the year are gathered either monthly or quarterly and reported for individual units and hospital wide. For this project, data were obtained from the portal for falls, HAPIs, CAUTIs, and hand hygiene adherence. Hand hygiene data are collected monthly during mystery shopper rounds.

Press Ganey questionnaires typically use a 5-point Likert scale to measure patient experience, with each point reflecting the respondent's level of satisfaction. Possible item scores usually range from 1 (Very Poor) to 5 (Very Good), representing progressively more positive perceptions of care.^18^ The results are reported based on the scores from each question and certain sections are grouped for overall satisfaction, which is reflected as a percentage score. This project reports “Nurse Overall Satisfaction” percentage scores as indicators of patient satisfaction with how well their needs were met.

**Table:** Implemented actions including the domain and initiator

Implemented action	Domain	Initiator
Patient engagement and communication training from patient experience team	Patient satisfaction	Manager
Reduced sound of call bell volume	Patient satisfaction	Nurse UBC
Daily positive communication initiative. Modeling positive, friendly attitude, greeting each staff member as well as patients and visitors.	Nurse satisfaction	Manager
Flexible duty schedule practices including two requests per month	Nurse satisfaction	Nurse UBC
Fair rotation of charge nurses when two are scheduled on same shift	Nurse satisfaction	Manager
Multidisciplinary team morning huddles to include hand hygiene awareness	Clinical outcomes	Nurse UBC
Creation of teams for NSQIs falls, HAPI, hand hygiene, CAUTI. Each team has four members led by a charge nurse and all members contribute to compliance audits and provide awareness sessions in huddles.	Nurse satisfaction and clinical outcomes	Manager
Daily status exchange huddles after physician rounds	Nurse satisfaction and clinical outcomes	Nursing model of care
Nurse leader clinical rounds checklist to include post round discussion of findings with the whole team	Nurse satisfaction, patient satisfaction, and clinical outcomes	UBC and manager

The NDNQI RN Satisfaction Survey is a questionnaire that provides satisfaction scores for individual items as well as aggregated section scores.^18^ The scores reflect the Likert-type scale format, where questions are asked within categories and answer options are strongly agree, agree, neutral, disagree, and strongly disagree.^17^ The raw mean scores are converted into a standardized metric.^17^ For this project, the authors analyzed the overall section scores for “Supportive Manager” in the acute stroke unit.

### Analysis

Descriptive analysis was conducted on all data collected for the clinical outcomes and patient satisfaction. The clinical outcome data are published monthly and presented in the form of bar and run charts and described according to the level of measurement. This format allows for inferences to be drawn about the effectiveness of the strategies aimed at improving clinical outcomes and patient satisfaction.^15^,^20^ The RN satisfaction survey is conducted annually, and statistical analysis is performed by the ANCC then reported to the hospital.^17^ The survey was conducted in December 2024 preimplementation and then in June 2025, 6 months postimplementation. The two scores were compared and inferences made about the effectiveness of interventions aimed at improving nurses' satisfaction.

## Results

The nurse manager obtained the monthly, quarterly, and annual data and tracked the progress across the three domains of clinical outcomes, patient satisfaction, and nurse satisfaction. The RN satisfaction survey was completed by all 26 nurses working in the acute stroke unit.

Press Ganey Patient Satisfaction Survey results demonstrated progressive improvement across 2025 compared with 2024 baseline data (see *Press Ganey Patient Satisfaction Survey scores for “overall nursing,” expressed in percentages*). Scores in the nursing domain increased each quarter, reflecting improved patient perceptions of nursing care quality and responsiveness. The most notable gains were observed in Quarters 2 and 3, suggesting sustained impact of the interventions implemented.

Hand hygiene audits revealed marked variation in adherence rates over time (see *Hand hygiene technique adherence scores*). Adherence decreased from 80% in 2024 to 64% in Quarter 1 2025, indicating an initial decline following the project rollout. However, utilizing PDSA processes to develop targeted reeducation and reinforcement strategies was associated with a strong rebound. Adherence returned to 80% in Quarter 2 and further improved to 87% in Quarter 3 2025. This 7% increase compared with the baseline demonstrates the effectiveness of continuous monitoring and feedback, which is at the core of PDSA.^13^

Staff satisfaction, as measured by the NDNQI RN Satisfaction Survey, improved across all measured domains between 2024 and 2025 (see *NDNQI RN Satisfaction Survey results for the stroke unit in 2024 and 2025*). Ratings for “Supportive Manager” increased most significantly, from 4.42 to 5.30, reflecting positive perceptions of leadership. “Autonomy,” “Job Enjoyment,” and “Decision Making” also all increased over time. These results suggest that interventions enhanced the professional practice environment, with leadership support emerging as the strongest driver of improvement.

Both falls with injury and HAPIs stage 2 and higher increased during the roll-out phase in the first quarter of 2025. Falls with injury occurred at a rate of 2.08 per 1000 patient days in Quarter 1 2025, an increase from 0.10 in 2024 (see *Incidence of falls with injury per 1000 patient days from Q4 2024 to Q3 2025*). HAPIs stage 2 and higher occurred at a rate of 4.59 per 1000 patient days in Quarter 1 2025 then decreased to 1.83 in Quarter 2 2025 (see *Incidence of HAPIs stage 2 and higher per 1000 patient days from Q4 2024 to Q3 2025*). There have been no incidents of falls or HAPIs in Quarter 3 2025. The incidence of CAUTIs was unchanged (0 per 766 catheter days in 2024 and 0 per 452 catheter days in 2025).

**Figure:**
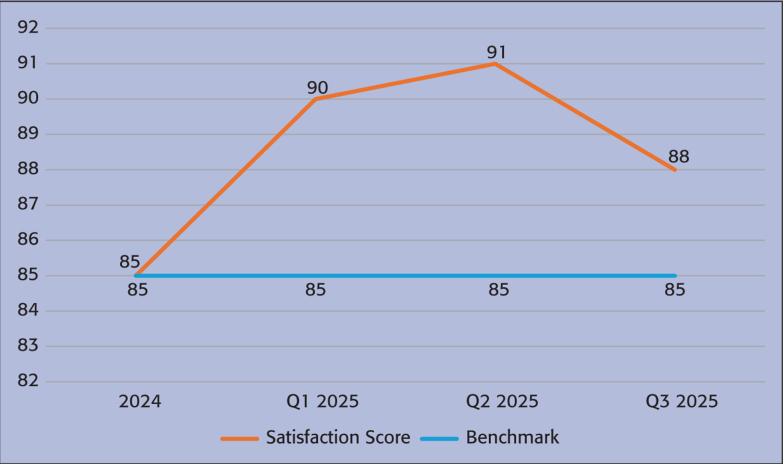
Press Ganey Patient Satisfaction Survey scores for “overall nursing,” expressed in percentages

**Figure:**
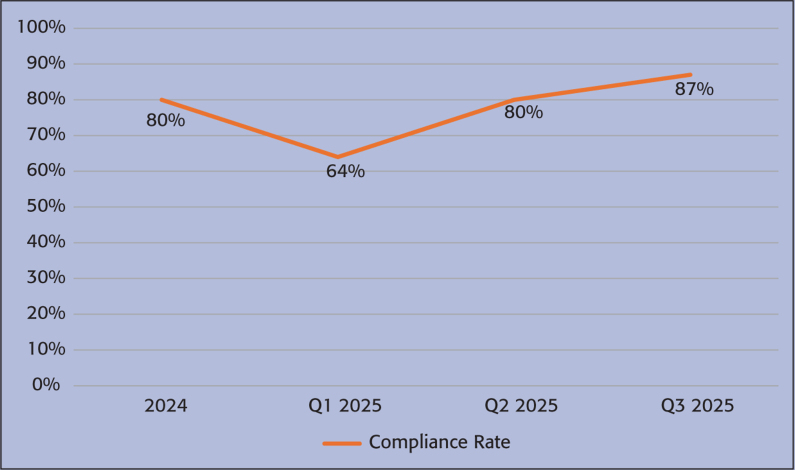
Hand hygiene technique adherence scores

Percentages determined by mystery shopper audits from 2024 to Quarter 3 2025.

During Quarter 1 of 2025, the unit expanded capacity by opening five additional beds, increasing the total from 20 to 25. However, nursing staff numbers were not adjusted to reflect this increase until the end of March. Concurrently, the unit admitted internal medicine outlier patients presenting with multiple comorbidities and elevated care requirements, in response to a hospital-wide surge in patient volumes. Although a direct causal relationship could not be empirically established, the data indicate a potential correlation. Notably, following the adjustment in staffing levels, hand hygiene adherence improved and no further incidents of patient falls or HAPIs were reported through the end of Quarter 3, 2025.

**Figure:**
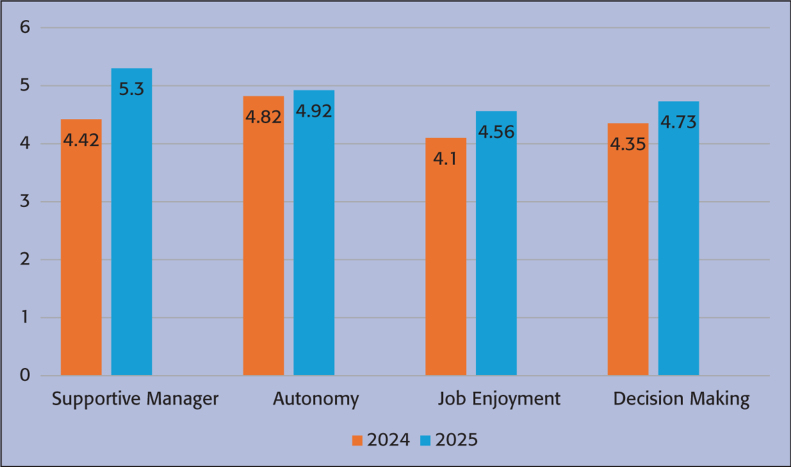
NDNQI RN Satisfaction Survey results for the stroke unit in 2024 and 2025

**Figure:**
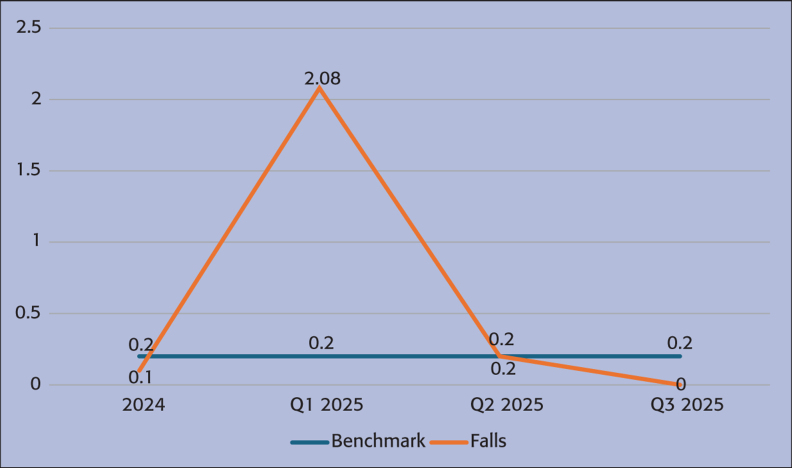
Incidence of falls with injury per 1000 patient days from Q4 2024 to Q3 2025

## Discussion

This QI project demonstrated that evidence-based interventions can significantly improve nurse satisfaction, patient satisfaction, and clinical outcomes within an acute stroke unit. The results highlight the relationship between workforce support, leadership, and patient safety outcomes and directly address the project's aim of improving both staff and patient experiences while maintaining cost-effectiveness and sustainability.

Acute stroke units face unique challenges when addressing HAPIs, falls, patient satisfaction, and nurse satisfaction. Patients frequently present with comorbidities such as diabetes mellitus and mobility restrictions due to impairment that significantly increase their HAPI risk.^21^ Cognitive impairment and confusion are prevalent following stroke, increasing fall risk through nonadherence with safety protocols and/or memory impairment.^22^ Communication barriers arise from neurologic injury to language centers, and multinational population demographics create additional communication challenges between patients and nursing staff.^23^ The intensive nursing resources required to provide this high level of care strain workforce capacity and engagement. Therefore, systematic, evidence-based approaches must address the interconnectedness of clinical outcomes and staff satisfaction while remaining cost-effective and sustainable.

A key finding was the marked improvement in patient satisfaction, particularly within the nursing domain, where scores consistently increased across 2025. These results suggest that interventions such as enhanced patient communication training, daily nurse leader clinical rounding, and structured huddles enabled more responsive, person-centered care. Similar results have been reported in several previous studies conducted internationally, including in the UAE.^4^-^6^,^12^

Staff satisfaction also improved across multiple domains, with the largest gains seen in perceptions of supportive leadership. These results affirm the effectiveness of transformational leadership and shared governance in cultivating a more positive practice environment and highlight leadership support as a critical driver of engagement.^6^,^8^,^10^,^24^

Regarding clinical outcomes, hand hygiene adherence initially decreased but rebounded strongly following targeted education and reinforcement, ultimately surpassing baseline performance. Again, these results align with the findings of previous research on hand hygiene adherence, including a systematic review.^25^,^26^ This pattern highlights the importance of continuous feedback and monitoring in sustaining behavioral change facilitated by the PDSA model.^3^,^8^,^11^,^13^,^27^ Notably, although falls with injury and HAPI incidents spiked during Quarter 1, these coincided with the expansion of bed capacity without a corresponding increase in nursing staff and the admission of high-acuity internal medicine patients. Once staffing was increased to match the workload, no further falls or HAPI events occurred through Quarter 3. Although causality cannot be established, the association between adequate staffing levels and patient safety outcomes is compelling. These results also echo the findings of other research studies.^12^,^28^

The impact of this initiative extends beyond individual metrics. For patients, the outcomes translated into improved safety and satisfaction with nursing care. For staff, the project fostered greater autonomy, empowerment, and trust in leadership, which are known to support retention and job satisfaction.^4^,^6^,^29^,^30^ At the organizational level, the results demonstrate the potential for systemwide benefits when investment is made in leadership development and workforce capacity, contributing to the broader institutional goals of Magnet Recognition^®^ and high-reliability care delivery.^17^

**Figure:**
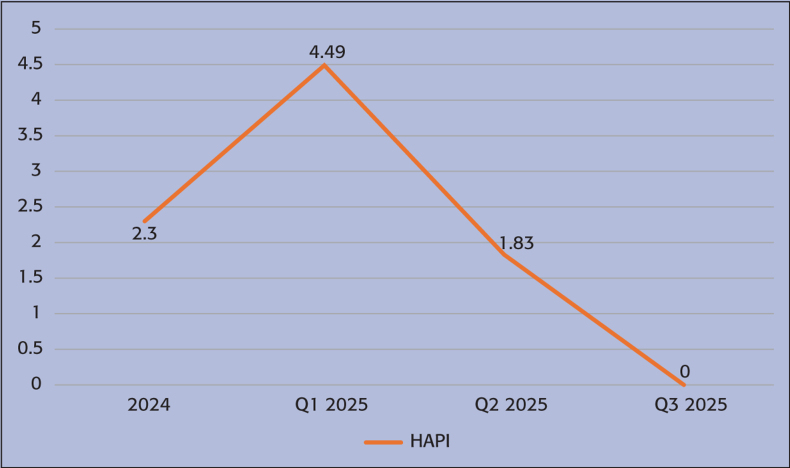
Incidence of HAPIs stage 2 and higher per 1000 patient days from Q4 2024 to Q3 2025

### Strengths and limitations

There were several strengths that underpinned the project's success. The use of transformational leadership and shared governance frameworks along with the PDSA model ensured a structured, theory-informed approach.^27^,^31^,^32^ Staff engagement was maximized through team-based ownership of quality indicators and opportunities for frontline staff to contribute via the DTS.^3^,^7^,^8^,^33^ The team leaders were empowered to allocate tasks such as auditing and unit in-service session development to all team members. As a result, work was evenly distributed within the team, reducing the burden on any one individual, particularly the leader. The manager maintained responsibility for collating and reporting audit data and attendance records. Furthermore, the integration of multidisciplinary collaboration, flexible rostering, and equitable workload allocation contributed to improved safety outcomes as well as higher nurse morale and professional fulfilment. Staff who demonstrated engagement and participation that exceeded expectations were formally recognized through the hospital recognition program, “Witnessing Outstanding Work” which included awarding certificates and vouchers.

In terms of limitations, this QI project was conducted within a single specialized unit of a tertiary hospital, which may limit the generalizability of findings to other clinical areas with different patient populations, resources, or organizational cultures.^20^ In addition, although associations were observed between interventions and outcomes, the project design did not allow for statistical testing of causality, and improvements may have been influenced by unmeasured variables. For example, broader hospital-wide initiatives or seasonal fluctuations in patient acuity may have contributed to outcome variation.

### Implications

The outcomes have important implications for nursing practice, patient care, and organizational strategy. The reported improvements in patient satisfaction, staff engagement, and clinical outcomes highlight the value of investing in leadership development, shared governance, and structured QI frameworks to drive sustainable transformation. The findings reinforce the critical link between adequate staffing levels, supportive leadership, and patient safety, highlighting the need for health care organizations to align workforce capacity with patient acuity to prevent adverse events.^1^,^8^,^24^ These results suggest that similar evidence-based, staff-driven approaches can be scaled across other clinical areas to strengthen safety culture, enhance workforce retention, and advance institutional goals such as Magnet Recognition^®^ and high-reliability care.

## Conclusion

This QI project demonstrated that structured, evidence-based strategies grounded in transformational leadership, shared governance, and QI frameworks can significantly enhance patient satisfaction, nurse engagement, and clinical outcomes within an acute stroke care setting. The findings highlight the pivotal role of supportive leadership and adequate staffing in ensuring safe, person-centered care, while also showing the value of empowering frontline nurses to lead and sustain quality initiatives. Although conducted in a single specialized stroke unit, the outcomes provide a practical model that can be adapted across other clinical areas, supporting organizational goals such as Magnet Recognition^®^ and advancing a culture of safety, accountability, and continuous improvement. Future work should focus on evaluating the scalability of these interventions across multiple units and care settings to determine their broader applicability. Incorporating more robust scientific research methods, such as experimental or mixed-methods approaches, may provide stronger evidence of causality and capture the impact of leadership and cultural change. Further exploration of long-term sustainability will also be critical, particularly in ensuring that nurse engagement, patient satisfaction, and clinical outcomes remain stable.
